# Advancing Nursing Data Integration Through a Nursing Minimum Dataset for the Conceptual and Technical Development of a “Fall Prevention” Data Module: Development Study

**DOI:** 10.2196/82417

**Published:** 2026-03-17

**Authors:** Sarah Milkov, Antonia Schmidt, Anja Burmann, Niklas Tschorn, Marcel Klötgen, Wolfgang Deiters, Christian Potthoff, Kirsten Neveling, Yvonne Weber, Maren Keuchel, Daniela Holle

**Affiliations:** 1 Hochschule Bochum Bochum Germany; 2 Fraunhofer Institute for Software and Systems Engineering ISST Dortmund Germany; 3 Fraunhofer Institute for Software and Systems Engineering Dortmund, North Rhine-Westphalia Germany; 4 Diakonie Michaelshoven Pflege und Wohnen gGmbH Köln Germany; 5 Connext Communication GmbH Paderborn Germany

**Keywords:** nursing, minimum dataset, core dataset, fall, standardization, decision-making

## Abstract

**Background:**

In aging populations, the demand for care, including care delivery in long-term care (LTC) facilities, is increasing. This situation highlights the need to optimize care processes through continuous scientific evaluation. The use of artificial intelligence (AI) has the potential for use in nursing research, but it experiences a lack of standardization and structuring of nursing data. Although solutions such as standardized nursing terminologies exist, their use in practice has thus far not been widespread and is often associated with high documentation costs.

**Objective:**

This paper presents the conceptual and technical development of a nursing minimum dataset that focuses on a specific “fall prevention” use case. The aim of this work was to improve data standardization and usability for research and AI-based analysis in LTC settings.

**Methods:**

A representation of the “fall prevention” use case was developed using literature analyses, co-design workshops, and a quantitative survey (n=158). Technical indexing was conducted by translating the results into the technical terminology of the Health Level Seven International Fast Healthcare Interoperability Resources standard.

**Results:**

The “fall prevention” use case was developed as part of a German nursing minimum dataset for long-term residential care with 8 basic modules (patient or client demographics) and 11 extension modules (nursing care elements). The module of the “fall prevention” use case includes fall risk factors, interventions, and outcomes. The literature analysis included 4 international fall guidelines and 17 practice and transfer documents established in German LTC. In total, 12 experts from the fields of management, quality management, technical application support, nursing service management, department management, and members of the PFLIP (Pflege-Kerndatensatz und Intersektorales Pflegedaten-Repository [Nursing Minimum Data Set and Intersectoral Nursing Data Repository]) research project participated in the workshops. A total of 158 people participated in the quantitative survey, the majority of whom were female (117/158, 74%), with 63% (100/158) working directly in nursing care and an average of 24.9 years of professional experience, mainly in LTC (63/158, 40%), outpatient care (37/158, 23%), and hospitals (14/158, 9%). The relevant content, in the sense of a minimum set of items, was identified and prioritized in collaboration with nursing experts and translated into a Fast Healthcare Interoperability Resources–based implementation guide.

**Conclusions:**

This approach addresses the lack of structured nursing data for AI and research and can serve as an example for interoperable, cross-sector solutions in global LTC.

## Introduction

### Background

Demographic change in Germany and Europe is leading to a steadily growing number of people in need of care, especially older people with multiple health problems [1]. A growing problem in this age group is falls, which place considerable physical and psychological strain on both those affected and the care systems and increase the need for treatment [2,3]. The continuous scientific development of nursing processes is essential for improving the quality of care, promoting evidence-based knowledge, and further developing nursing practices [4,5].

**Figure 1:**
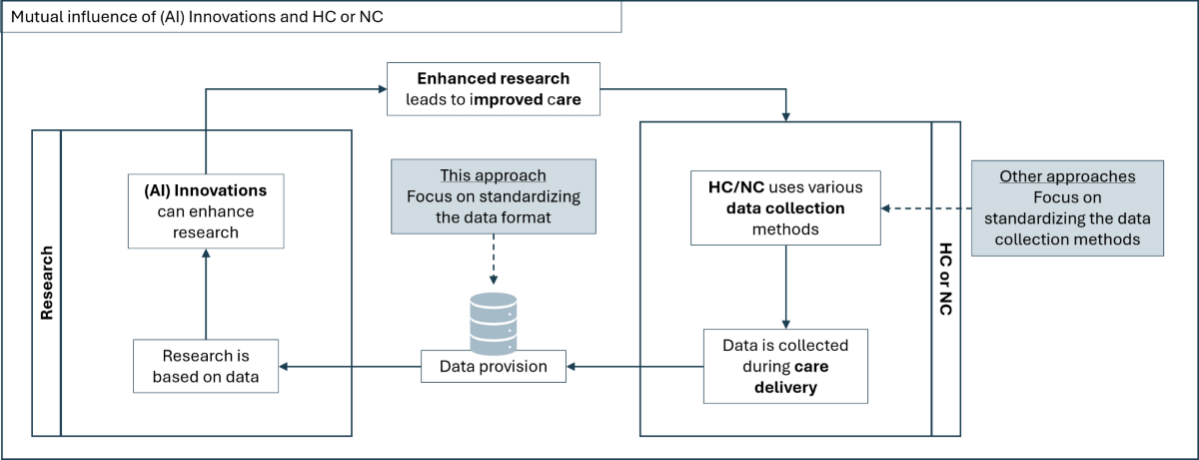
Nursing research, care, and AI innovation cycle. AI: artificial intelligence; HC: health care; NC: nursing care.

The application of artificial intelligence (AI) provides new possibilities for nursing research by enabling the efficient analysis of large amounts of care data, recognizing patterns, and obtaining scientifically useful insights [6]. This strengthened nursing research contributes significantly to improving and facilitating nursing care, as scientific findings can be used to develop evidence-based care strategies [7]. In turn, data from nursing care form the basis for AI-supported analyses (Figure 1) [8]. The cyclical process between (AI-based) research, health care and nursing care, and AI innovations, with data serving as the central interface, is shown in Figure 1. Using the example of fall prevention, AI innovations can enhance research opportunities, such as studies on falls in long-term care (LTC). The findings from this research can then improve the quality of nursing care and help prevent falls. In nursing care, data are continuously generated, for example, when falls are documented, which feeds back into research and supports further AI-driven innovations in fall prevention. The challenge is to bring the data collected in the health care system into a uniform format that is accessible for research.

The secondary use of existing documentation data offers an alternative to traditional prospective studies for research purposes. However, accessing and harmonizing these data are challenging due to heterogeneous systems and varying documentation practices, which often result in unstructured or nonstandardized data.

Existing solutions, such as internationally recognized nursing terminologies or so-called standardized nursing languages, aim to standardize the documentation of nursing data by specifying certain terms, topics, and terminologies [9]. However, the use of standardized nursing terminology and specialist languages is not common across Europe. The use of structured nursing documentation varies widely across countries, and studies by Thoroddsen et al [10] and Dos Santos et al [11] suggest that nursing diagnoses and interventions are used most frequently.

The general use of standardized nursing terminology is still lacking, which makes access to nursing data more difficult [10]. A cross-sectional study from 2020 in the Netherlands revealed that approximately half of the 667 nurses and assistants surveyed would use standardized terminology in electronic documentation [12]. The reasons for this include burdensome documentation (use barrier) and a still predominant use of medical terminology (terminology barrier), although medical terminology is insufficient for describing nursing processes, such as assessing unsteadiness or unsuitable footwear. Other studies also show that complex documentation is inefficient in care practice, consumes resources necessary to perform care measures, and leads to a considerable documentation burden for nursing staff [11,13-16].

In the case of Germany, LTC facilities took a step away from standardization toward more patient-oriented documentation in 2015 with the introduction of a law to reduce bureaucracy in care documentation, which led to an increased use of unstructured free-text entries [17,18]. The absence of legal provisions governing the use of standardized terminology constitutes a structural barrier to the implementation of such concepts [19]. Furthermore, there is currently a paucity of terminology that has been specifically developed for German LTC, with the literature consisting of translations of foreign approaches. Nursing care systems are characterized by a high degree of heterogeneity in different countries, which reinforces the use barrier [20].

This paper addresses a research gap in German LTC caused by the lack of integration and routine use of nursing terminology. This situation gives rise to a paucity of structured and standardized data, while existing approaches to data use remain ineffective, resulting in the untapped potential of valuable data for research purposes remaining unused. Our approach aims to standardize the format of research data rather than the methods of data collection, allowing nursing staff to continue working as usual, while their collected data are converted for research purposes (Figure 1).

The following research questions are addressed:

- What conceptual, nursing, and technical requirements are necessary to develop a nursing minimum dataset (NMDS) based on existing nursing care data from routine documentation using the example of the “fall prevention” use case?
- How can a minimum modular nursing dataset contribute to the harmonization and integration of nursing data for cross-sectoral care and data-based research?

The conceptual and technical development of an NMDS according to the definition by Werley et al [21] for the context of LTC is presented via the “fall prevention” use case, and its potential is discussed in terms of the challenges mentioned. In our use case, an analysis is conducted of data on risk factors, interventions, and outcomes of falls with the aim of preventing such incidents. In addition, a possible practical application in the context of the AI-supported analysis of nursing data is presented.

### Objectives

This paper presents a new approach to enable the secondary use of nursing data without additional effort in nursing practice. To this end, the following objectives are pursued: (1) outline the necessary steps for the development of an NMDS based on the “fall prevention” use case, guided by the literature and expert input; (2) describe the technical approach to systematically record fall risk factors, including the collection and prioritization of key factors; and (3) provide a perspective on an NMDS for data collection and harmonization, demonstrating its applicability for research and AI-based analysis.

### Related Work

Some related work must be accounted for when addressing the research objectives. The German research project PFLIP (Pflege-Kerndatensatz und Intersektorales Pflegedaten-Repository [Nursing Minimum Data Set and Intersectoral Nursing Data Repository]), on which the following results rely, aims to standardize previously collected nursing data to make the data available for research and AI applications and to offer the possibility of harmonizing data across sectors [22]. This project is based on the German Medical Informatics Initiative (MII), which has developed a medical core dataset on which the NMDS presented in this paper is based [22,23].

With respect to the scientific foundations of fall risk assessment, the “fall prevention expert standard” guidelines served as a foundation for the development of a use case. In Germany, the nursing guidelines that define best practices and quality benchmarks to ensure high standards of nursing care are called “expert standards” [2].

With respect to standardization and interoperability, the National Association of Statutory Health Insurance Physicians in Germany publishes relevant health care transition documents for interoperable data exchange in a telematic infrastructure. The work of this organization is used as a basis for the development of conceptual and technical use cases [24].

To enable standardized fall data to be used on a broader basis, a suitable data format must be chosen. The Health Level Seven International *(HL7)* Fast Healthcare Interoperability Resources *(*FHIR*) standard* is an internationally recognized framework for the exchange of health information, and its technical terminology is used by health care providers, software developers, and governments worldwide to improve interoperability between different systems and facilitate access to health data. The active community of FHIR includes professionals from various health care fields, who work together to further develop and implement the standard to optimize global health care.

This paper examines the development of an NMDS for LTCs using the example of the “fall prevention” use case. Building on initiatives such as the MII, German expert standards, and international standards such as HL7 FHIR, the NMDS aims to harmonize nursing data, integrate it across sectors, and make it usable for both practice and research, assuming that such an approach is feasible, relevant to practice, and sustainable to implement.

## Methods

### Overview

**Figure 2:**
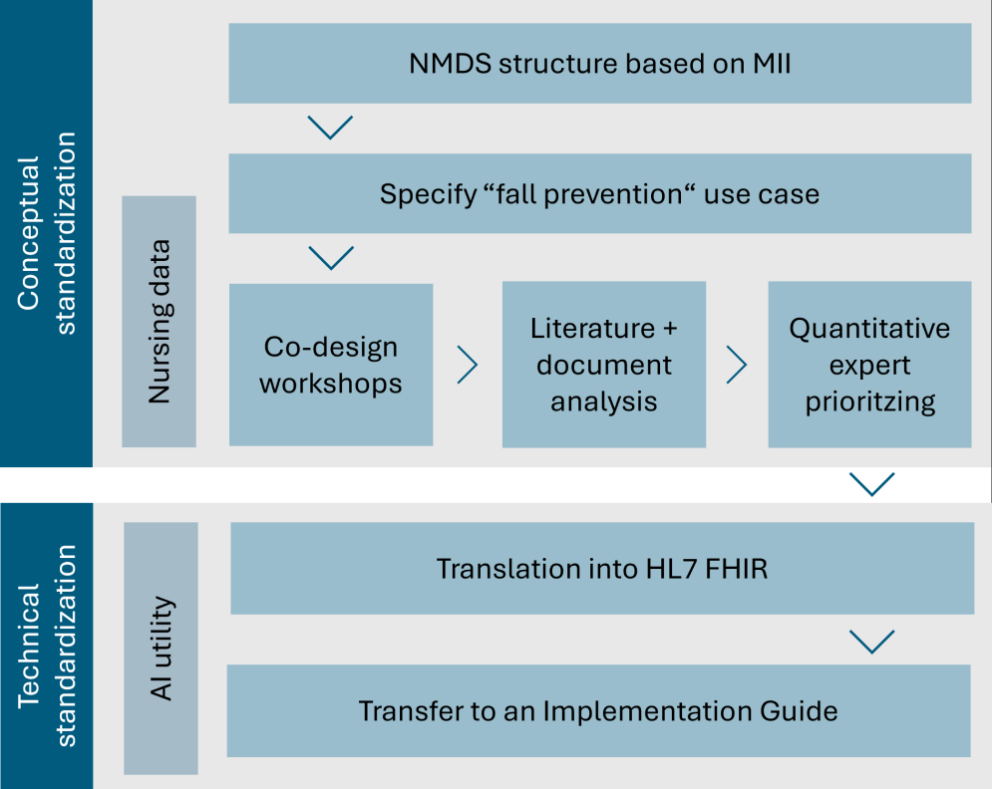
Methodological approach. AI: artificial intelligence; FHIR: Fast Healthcare Interoperability Resources; HL7: Health Level Seven International; MII: Medical Informatics Initiative; NMDS: nursing minimum dataset.

The methodology for achieving our research goals consists of an approach of conceptual standardization, which we combine with and translate into technical standardization. An overview of the procedure is presented in Figure 2.

### Conceptual Standardization

#### Overview

A co-design approach was used to develop the content of the “fall prevention” use case of an NMDS. Co-design, according to Sanders and Stappers [25], is an approach that describes collective creativity throughout an entire design process. Designers collaborate to develop innovative solutions by integrating diverse perspectives and skills into the design process [22]. Within this approach, an NMDS structure was developed, and the use case was selected and developed in a participatory manner. The development was supplemented by literature and document analyses and a quantitative survey of nursing staff, and the initially broad approach was narrowed down to a minimum set of items.

#### Co-Design Development of an NMDS Based on the “Fall Prevention” Use Case

The aim was to design a structure for an NMDS and specify the “fall prevention” use case. The use case was considered particularly suitable because a fall event is extensively documented for insurance-related reasons; therefore, extensive data are available [2].

The foundation for this work was a model developed within the PFLIP research project, drawing on the MII medical core dataset and the German guideline “Expert Standard of Nursing,” which served as a basis for this study, while details of its development are not reported here (Multimedia Appendix 1) [20,23]. A qualitative, participatory design in the form of a co-designed expert workshop was chosen to achieve the stated goal.

Participants were recruited through the management of the participating nursing facility in writing and through personal contact. The sample was specifically selected to reflect a range of nursing expertise, and the criteria for participation were work in the professional fields of general management, quality management, technical application support, nursing service management, nursing department management, and contact with fall documentation in everyday work. No restrictions were imposed regarding sociodemographic data. Before the workshop took place, all the participants provided their consent for the procedure and data processing. Participation was voluntary and could be discontinued or revoked at any time. The data were processed anonymously; thus, no conclusions could be drawn about individuals, and the data were handled in accordance with the General Data Protection Regulation.

The study was not approved by an institutional review board. The aim of the study was to gather opinions and assessments from employees of a nursing facility as experts on a fictitious use case. This is not interventional or experimental research on humans, and no personal data were collected.

The “fall prevention expert standard” [26] served as the foundation for the use case and was initially evaluated for completeness by the nursing staff during a workshop; its content was visualized for the participants, which allowed them to prioritize the listed fall-related items according to perceived relevance and to suggest additional aspects they considered important in the context of fall prevention. The results were compiled verbally and documented in writing by the research team. Additionally, patient journey mapping [27] was developed to understand how residents of nursing facilities navigate the nursing system, to illustrate the interfaces in the cross-sectoral documentation of a fall, and to identify additional documentation tools that provide information on the current standard fall documentation.

The results were evaluated using qualitative content analysis according to Mayring [28]. Coding was performed deductively using predefined categories derived from the expert standard. This approach enables a structured and comprehensible analysis of the data.

#### Literature and Document Search

Additional international guidelines for fall events were researched on the platform Google Scholar and in national guideline registers. Practice documents and legal requirements were evaluated to identify current documentation of falls in nursing care. The documents were searched directly on the website of providers for transfer documents in medicine and nursing care as well as an LTC facility in Germany. Finally, a targeted search for standardized nursing terminology was conducted to incorporate international standards. This search included an analysis of documents on the International Classification of Nursing Practice [29].

Guidelines that addressed the topic of “fall prevention” in LTC settings and among older adults were included. The guidelines were subsequently analyzed in terms of content (risk factors, interventions, and outcomes). All the documents were screened according to these criteria.

Relevant information from the guidelines and documents was systematically extracted and organized according to predefined categories of risk factors, interventions, and outcomes and presented in tabular form, ensuring that the guideline-based requirements relating to falls in LTC were clearly and comprehensively presented.

#### Quantitative Online Survey

An anonymous online survey was conducted to specify broad findings in a practice-relevant manner. Below, we report on the study following the CHERRIES (Checklist for Reporting Results of Internet E-Surveys) checklist [30].

The target population included people working in the nursing sector who were chosen for open convenience sampling. The questionnaire was distributed nationwide to nursing facilities in various settings as well as to nursing staff with different professional backgrounds and experience levels.

The survey was created with the software LimeSurvey (LimeSurvey GmbH) and can be found in Multimedia Appendix 2. The aim of the questionnaire was to prioritize fall events identified by nursing staff. The questionnaire included demographic questions and a Likert scale on which the participants rated the relevance of various fall-related items (risk factors, interventions, and outcomes). The participants also indicated whether the items mentioned had previously been documented in their care facility and, if so, whether they had been documented digitally. The findings from the previous steps were used as a base set of questions for the survey.

The survey responses were descriptively analyzed and graphically presented via the software Looker Studio (Google LLC). As the survey was created in LimeSurvey, it was possible to set it up so that no incomplete questionnaires were saved; this ensured that no incomplete answers were included in the evaluation.

### Technical Standardization

In the next step, the interim results were translated into the technical terminology of the HL7 FHIR standard and made available for use in digital applications and AI-based systems.

#### Systematic Capture of Fall Risk Factors and Outcomes

To enable broader use of the standardized fall data, HL7 FHIR was chosen as a suitable data format. The methodology used to translate the identified fall parameters into HL7 FHIR involved a series of systematic processes. The focus was initially limited to fall risks that were documented digitally in our cooperating LTC facility and received more than 80% agreement on their relevance for fall detection by the nursing staff, as rated in the Quantitative Online Survey section. This restriction, including the deliberate exclusion of interventions, was made to conserve time- and effort-related resources. Initially, a thorough screening of the FHIR base resources was conducted to determine which resources were most suitable for representing the identified fall risk factors. Subsequently, existing implementation guides (IGs), such as the MII core dataset, were searched for eligible profiles that could be used as a basis for further profiling. Fall risk factors and fall outcomes were subsequently mapped to appropriate codes in the Systematized Nomenclature of Medicine Clinical Terms [31] and the *ICD-10* (*International Statistical Classification of Diseases and Related Health Problems*) [32] to ensure that each risk factor was accurately represented in a standardized format that can be widely recognized and used in the health care industry. An example of this is shown in Multimedia Appendix 3. Finally, FHIR Shorthand was used during the profiling process to create a concise, human-readable, and computable representation of the mapped fall risk factors; this significantly simplified the process of creating and maintaining FHIR resources and profiles.

#### Perspective on a “Fall Prevention” NMDS for Data Collection and the Harmonization of Nursing Data for Research Purposes

In addition to the systematic capture of fall risk factors, the definition of “fall” NMDS is an important part of the reusability of the data and its structure. As discussed in the previous sections, this secondary use of data plays an essential role in research on health services. These aspects are also recognized by research data networks such as the German MII, which relies on FHIR. To be compatible with these structures in the future, the NMDS “fall” use case for data collection and the harmonization of nursing data were transformed to FHIR.

An IG was created that provides a detailed, step-by-step manual that effectively communicates how to implement the mapped fall risk factors in FHIR. The IG offers a topic-specific bundled presentation of profiles, value sets, and other FHIR data structures. The need for this IG is based on recognition of the complexity of health care data and the challenges that can arise when standardized health care data models such as FHIR are implemented.

Another argument in favor of following the example of existing research data networks is the prospect of being connected to them. Considering and incorporating the FHIR-based core dataset of the MII ensure that the collection of fall risk factors can be included in the research data network and made available to researchers.

### Ethical Considerations

This study did not require approval by an institutional review board, as it did not involve interventional or experimental research with human participants. The study aimed to collect opinions and assessments from nurses as experts regarding fictitious fall risks derived from the literature. All participants received written information about the purpose, scope, and procedures of the questionnaire. Participation was entirely voluntary, and informed consent was obtained prior to data collection. Participants were informed that their data would be processed anonymously. The study was conducted in accordance with the principles of the Declaration of Helsinki, the guidelines of the Council for International Organizations of Medical Sciences, the World Health Organization, the European General Data Protection Regulation, the German Federal Data Protection Act, and the Data Protection Act of the State of North Rhine-Westphalia (Landesdatenschutzgesetz NRW). As all survey data were fully anonymized and no information was collected that would allow the identification of individual participants, the study does not involve the processing of personal data as defined in Article 4(1) of the European General Data Protection Regulation. All data were stored on protected servers. All participating institutions approved the study as part of a data protection agreement, with the involvement of data protection experts.

## Results

### Conceptual Standardization

The “fall prevention” use case was conceptually developed on the basis of an NMDS based on the MII core data (Multimedia Appendix 2).

#### Co-Design Development of an NMDS Based on the “Fall Prevention” Use Case

The workshops took place in August 2022. In total, 12 participants from the fields of management, quality management, technical application support, nursing service management, and department management and members of the PFLIP research project participated in the co-design workshops. Experts from the field of nursing science also played a key role in developing patient journey mapping. During this process, participants observed that while the expert standard provides valuable insights, it could be further enriched by considering additional factors to capture the full complexity of falls, leading to an expansion of the topic. Patient journey mapping helps to identify critical interfaces, relevant documentation, and transfer forms that are essential for a comprehensive representation of fall-related processes. The results are presented in Multimedia Appendix 4. On this basis, a search for these documents was initiated to incorporate them into the development of the use case.

#### Literature and Document Search

As part of the literature analysis, 4 additional international fall guidelines, 5 documents that are routinely applied in German LTC practice, 6 documents from a provider of transfer documents in Germany, 6 transfer documents from a selected LTC facility in Germany, and the International Classification of Nursing Practice falls catalogue were included. Many of these documents depict fall events rather generally. Thus far, no study has comprehensively addressed falls along with associated nursing-relevant risk factors and intervention options.

#### Quantitative Survey

The quantitative online survey was conducted from March 17, 2023, to July 30, 2023, with a total of 158 participants, 117 of 158 (74%) of whom were identified as female. Among these participants, 63% (100/158) were directly involved in nursing care. The participants had an average of 24.86 years of professional experience, ranging from 0.5 to 45 years. Most respondents (63/158, 40%) worked in LTC at the time of the survey, followed by home care (37/158, 23%) and hospital settings (14/158, 9%).

Risk factors were identified as items in the following categories: diseases, disease-related changes, environment, medications, and other factors. As interventions, items were identified in the following categories: physical measures, clothing adjustment measures, environmental adjustment measures, training and counseling measures, measures involving other professional groups, measures for the use of assistive devices for fall and injury prevention, the use of technology-based measures, and additional nursing measures. As outcomes, items were identified in the category of information about fall events. The results, including the identified content of the “fall prevention” use case, are presented in Multimedia Appendix 5. Table 1 shows the prioritized risk factors, including the percentage of agreement, which were further processed in the subsequent steps.

**Table 1:** Prioritized fall risk factors.

Risk factor	Rated importance, n (%)
1. Dizziness (vertigo)	158 (100)
2. Parkinson disease	158 (100)
3. Gait instability	158 (100)
4. Poststroke condition	157 (99)
5. History of falls	157 (99)
6. Overestimation of abilities	157 (99)
7. Mobility impairment	156 (99)
8. Antidepressants	156 (99)
9. Multiple sclerosis	156 (99)
10. Musculoskeletal impairment	155 (98)
11. Assistive devices (eg, rollator)	155 (98)
12. Visual impairment	154 (97)
13. Delirium (confused state)	154 (97)
14. Blood pressure fluctuations	154 (97)
15. Joint paresis (paralysis)	153 (97)
16. Confusion	151 (96)
17. Dementia	151 (95)
18. Fear of falling	150 (95)
19. Cognitive impairment	149 (94)
20. Sensorimotor impairment	148 (94)
21. History of fractures	148 (94)
22. Polypharmacy	147 (93)
23. Multimorbidity (multiple conditions)	146 (92)
24. Blood sugar fluctuations	146 (92)
25. Inadequate activities	146 (92)
26. Compliance	145 (92)
27. Alcohol or drug or nicotine abuse	143 (90)
28. Limb amputation	142 (90)
29. Antihypertensives (blood pressure–lowering drugs)	142 (90)
30. Psychotropics (affecting the psyche)	140 (89)
31. Pain	140 (88)
32. Anticonvulsants (to prevent epileptic seizures)	139 (88)
33. Cardiac arrhythmia	137 (86)
34. Measures involving deprivation of liberty	136 (86)
35. Wandering behavior	133 (84)
36. Antiarrhythmic medications	131 (83)
37. Incontinence	130 (82)
38. Sleep disorder	127 (80)
39. Osteoarthritis	127 (80)

### Technical Standardization

#### Systematic Capture of Fall Risk Factors

An overview of the risk factors considered for translation into FHIR can be found in Table 1. The objectives were considered because these received more than 80% agreement on relevance and were currently documented in our cooperating LTC facility.

When the risk factors were translated into the appropriate FHIR resources, existing profiles were screened. For risk factors involving a disease diagnosis, it was necessary to distinguish between medical and nursing diagnoses, because the term “diagnosis” is legally protected and relevant for billing purposes in the German health care system. In total, 60% (n=21) of the risk factors can be mapped as diagnoses, of which 24% (n=10) are nursing diagnoses and 26% (n=11) are medical diagnoses, whereby a total of 4 risk factors may be specified as both medical and nursing diagnoses. For 21% (n=9) of the risk factors, a nursing observation profile was created, which can be used to describe an occurrence during normal nursing activities. A further 12% (n=5) could be mapped to the MII medication profile. More specific profiles were created for the remaining 14% (n=7) of the risk factors, as they had more precise requirements and required differentiation. For example, the risk factor for incontinence was divided into fecal and urinary incontinence, and the necessity of a decision was included for measures involving deprivation of liberty.

#### Perspective on a “Fall Prevention” NMDS for Data Collection and Harmonization of Nursing Data for Research Purposes

At the heart of the developed IG is a questionnaire, represented by the FHIR resource questionnaire and questionnaire response, which was specifically designed for the “fall prevention” use case. The guide serves as an essential tool for ensuring consistency and accuracy in the implementation process across different systems and platforms. The response form composed of these 2 FHIR resources allows for the systematic documentation of all fall-relevant risk factors. The IG offers a collection of FHIR resources to capture previously identified risk factors for a fall. The order of the questionnaire response items reflects the prioritization of risk factors from the survey (Table 1). In addition to profiles designed solely for one or a few other risk factors, 3 more comprehensive profiles have been developed that can depict a multitude of risk factors (Tables 2 and 3) [33].

**Table 2:** Self-defined profiles in the PFLIP^a^ implementation guide.

HL7^b^ FHIR^c^ basic resource	Profile designation
Bundle	Multi-Morbidity (original Multimorbidität)
Condition	Medical-Diagnosis (original Medizinische-Diagnose) Nursing-Diagnosis (original Pflege-Diagnose)
Observation	Nursing-Observation (original Pflege-Beobachtung) Nursing-Observation-Urinary-Incontinence (original Pflege-Beobachtung-Harn-Inkontinenz) Nursing-Observation-Faecal-Incontinence (original Pflege-Beobachtung-Stuhl-Inkontinenz) Survey-Fall-History-6-Months (original Erhebung-Sturzhistorie-6-Monate) Deprivation-of-Liberty (original Freiheitsentziehende-Maßnahme)
Device	Aids (original Hilfsmittel)
List	Multi-Medication (original Multimedikation)
Questionnaire	Risk-Assessment (original Risikoermittlung)
QuestionnaireResponse	Risk-Assessment-Fall (original Risikoermittlung-Sturz)
Patient	Resident-Pseudonymized (original BewohnerIn-Pseudonymisiert)

^a^PFLIP: Pflege-Kerndatensatz und Intersektorales Pflegedaten-Repository (Nursing Minimum Data Set and Intersectoral Nursing Data Repository).

^b^HL7: Health Level Seven International.

^c^FHIR: Fast Healthcare Interoperability Resources.

**Table 3:** Extensions within the PFLIP^a^ implementation guide.

Name	Context of use
VoluntaryConsentExists (original *FreiwilligeEinwilligungLiegtVor*)	Deprivation-of-Liberty (original *Freiheitsentziehende-Maßnahme*)
CourtOrderExists (original *RichterlicheAnordnungLiegtVor*)	Deprivation-of-Liberty (original *Freiheitsentziehende-Maßnahme*)
DateOfBirth_mm_yyyy (original *Geburtstag_mm_yyyy*)	Resident-Pseudonymized (original *BewohnerIn-Pseudonymisiert*)
FullSurveyPeriod (original *VollständigerErhebungszeitraum*)	Survey-Fall-History-6-Months (original *Erhebung-Sturzhistorie-6-Monate*)

^a^PFLIP: Pflege-Kerndatensatz und Intersektorales Pflegedaten-Repository (Nursing Minimum Data Set and Intersectoral Nursing Data Repository).

## Discussion

### Principal Findings

The development of a German NMDS use case for fall prevention in LTC is based on a literature analysis of international guidelines and German practice documents, resulting in fall risk factors, interventions, and outcomes, and was developed in collaboration with 12 experts from various nursing fields. The relevant minimum items were prioritized by experts, reflected upon in a quantitative survey with 158 participants, and finally transferred into an FHIR-based IG.

The developed use case can benefit nursing research by making standardized, documented data available for secondary use and enabling AI-supported analyses. Since the dataset is based on routine documentation in nursing facilities and standardizes already documented data, it has the potential to support problems that have arisen thus far in efforts to standardize care documentation, such as a lack of manageability and the effort of documentation by nursing staff [13-16].

In contrast to broader international NMDS initiatives, the approach presented here takes a more detailed approach to a specific use case, resulting in practical, actionable information, better reflecting clinical variability, and creating a solid foundation for digital applications and AI.

In addition, the NMDS has been linked to the technical standardization HL7 FHIR from the outset, ensuring interoperability and direct technical implementability and guaranteeing connectivity to modern digital systems.

However, it should be noted that the dataset does not automatically ensure that documented data are also transferred to standards (such as FHIR). Data that are documented in other facilities and care documentation systems are not always automatically available in FHIR and may be in different formats. To counteract this limitation, a system solution, such as a data repository, is needed to automatically standardize routine data. The standardization of content and technology also facilitates the exchange of data between sectors and professions. In addition, the structural orientation toward an existing medical core dataset represents a step toward harmonizing nursing and medical data. This can support the need to give nursing terminology equivalent weight in documentation as medical terminology and integrate them instead of overwriting them [9,23].

Unlike previous approaches in LTC, which often failed to be implemented, the NMDS developed here is based exclusively on existing routine documentation to promote acceptance and long-term use in everyday nursing care without increasing the workload [34]. In accordance with extant recommendations in the literature, the development of NMDS should be undertaken in a multidisciplinary manner and integrated into (digital) documentation systems, with a focus on nursing content as opposed to medical content; thus, it should be oriented toward nursing standards, which we followed and built a base for [34,35].

The co-design approach chosen to develop the use case indicates a way of standardizing nursing data while maintaining a high degree of user centricity. This gives this NMDS an advantage over previous NMDSs and assessment instruments. In the future, a person-centered approach can be ensured by broadening the perspective to include affected persons, that is, residents of LTC facilities. The results in the NMDS table (Multimedia Appendix 5) illustrate the differences between risk factors that are perceived as relevant by practitioners and the scientific literature and those that are documented in a German geriatric care facility; this can indicate which important fall risk factors have not yet been mapped in routine documentation, such as “environmental factors” in this case. This information highlights the need for further research.

Although the results were collected nationally, findings from other European countries demonstrate difficulties in introducing standardized care documentation systems and standardization. This approach can serve as a model for the development of NMDSs for other countries [13-15].

There lies a challenge in bringing the data collected in the health care sector into a uniform format that is accessible for research. Once the data are accessible for research, AI innovations can benefit from a much larger data pool that is also very close to the point of care; this significantly improves the results of AI applications. The FHIR translation makes the data usable for AI applications that can assess and predict falls. The IG thus serves as a valuable resource for health care providers and researchers alike and provides a structured and standardized approach to capturing and analyzing fall risk factors. It is hoped that the future application of this guide will contribute to the development of more effective and personalized fall prevention strategies and ultimately improve patient outcomes. The developed IG also provides a methodological basis for expanding the minimum dataset to include other nursing topics in addition to falls as well as other care sectors and is internationally applicable.

### Proof of Concept: Practical Application Example—An AI-Based Nursing Decision Support System

An AI-based nursing decision support system (NDSS) for elderly people to predict falls in nursing homes can be used as an example of an application of the content and technology developed for our NMDS.

Developed in a user-centered design process based on International Organization for Standardization (ISO) 9241-210-2019, the NDSS covers routinely documented aspects of falls in German nursing homes and reflects the most relevant fall-related factors from the literature and practice [36]. Nursing staff with different levels of education and specialized training, administrative employees from an outpatient care facility and day care, and people who worked in the field of nursing science were asked about the practical suitability of the proposed NDSS in 4 workshops. Based on the workshop results, the NDSS was improved iteratively. The decision support system is designed to predict the risk of falling using a 5-step scale and focuses on the interpretability of AI so that predictions are comprehensible and transparent for users. Therefore, the NDSS can provide information about recognized risk factors that are responsible for corresponding fall risk. The proposed decision support system also suggests prophylaxis to reduce the risk of falling and show the progression of this risk over several months.

### Limitations

The results have several limitations. For reasons of feasibility, we focused on one use case. In this use case, we initially focused on a limited number of risk factors that were technically implemented. In the future, we plan to expand NMDS thematically. It must also be considered that there are different contexts, causes, and types of falls. Falls are presented in a highly standardized manner in this paper. In addition, to date, only aspects of inpatient LTC have been examined, but we plan to extend the findings to different settings.

It can be assumed that both the participants in the co-design workshops and the participants in the quantitative survey showed a certain level of interest in and affinity for the topic. As participation was voluntary, it is possible that less-interested participants did not take part due to participation bias.

Another potential bias in the quantitative study arises from the fact that participant evaluations were used as a criterion for item selection. Since documentation routines, levels of knowledge, and familiarity with existing evidence vary in everyday clinical practice, there is a risk that empirically relevant content will be underestimated or overlooked; this can lead to an overemphasis on subjective assessments, while evidence-based aspects remain potentially underrepresented. Moreover, we deliberately choose a user-centered approach to differentiate ourselves from previous solutions and ensure greater practical relevance. To reduce that bias, a broad survey covering a range of backgrounds and nursing staff was conducted. In the future, the perspectives of residents within the designated dataset should be incorporated. Furthermore, the participants’ ratings were based on international nursing standards and fall prevention guidelines and were not indiscriminately determined by the participants.

Nevertheless, it is believed that this approach is effective in enabling a user-centered methodology to be adopted, thus counteracting the lack of use and implementation of NMDS and nursing terminology and standards, as outlined in the problem statement in the introduction. It is important to validate the results in future studies.

The results are oriented toward national standards, practical procedures, and documentation in German LTC facilities. However, the procedure and methodology can be applied internationally.

### Conclusions

The “fall prevention” use case developed for a German NMDS in LTC shows how nursing data can be recorded in a standardized and user-centric way. The use of previously documented routine data reduces the documentation burden for nursing staff and facilitates cross-sector data exchange. This concept creates a valuable basis for nursing research by providing high-quality, structured data for secondary use. It also offers the potential for international application and can serve as a model for the development of nursing datasets in other subject areas, particularly through FHIR translation for use in AI-based applications.

The IG and the profiles it contains are to be submitted for an HL7 FHIR balloting. This structured mechanism is designed to solicit feedback from members of the HL7 community to ensure quality and relevance. The suggestions for changes and improvements collected in this process can be evaluated during further efforts and, if approved, can be integrated into the IG to promote the joint creation and further development of the NMDS.

In conclusion, our approach shows that co-design makes user-centered and applicable NMDS development feasible. Moreover, FHIR enables interoperability. This work represents a preliminary step toward harmonizing medical and nursing care in Germany by aligning the data format with MII structures, with future efforts needed to fully integrate the nursing sector. This paper enables us to adapt the approach internationally for AI-ready nursing data.

## Appendix

Multimedia Appendix 1 Nursing minimum dataset (NMDS). Visualization of the integration of an NMDS draft into the structures of the Medical Informatics Initiative (MII) core dataset as an extension module, whereby the MII core dataset is divided into basic and extension modules. The basic modules are defined in a cross-disciplinary manner. The extension modules map data from specific applications or specialist areas.

Multimedia Appendix 2 Online survey.

Multimedia Appendix 3 Example of assigning a fall risk factor to Fast Healthcare Interoperability Resources and Systematized Nomenclature of Medicine Clinical Terms codes.

Multimedia Appendix 4 Patient journey mapping.

Multimedia Appendix 5 Results: “fall prevention” use case.

## Abbreviations

AI: artificial intelligence
CHERRIES: Checklist for Reporting Results of Internet E-Surveys
FHIR: Fast Healthcare Interoperability Resources
HL7: Health Level Seven International
ICD-10: International Statistical Classification of Diseases and Related Health Problems
IG: implementation guide
ISO: International Organization for Standardization
LTC: long-term care
MII: Medical Informatics Initiative
NDSS: nursing decision-support system
NMDS: nursing minimum dataset
PFLIP: Pflege-Kerndatensatz und Intersektorales Pflegedaten-Repository (Nursing Minimum Data Set and Intersectoral Nursing Data Repository)

## Data Availability

The datasets generated during this study are not publicly available, as they were collected for internal project purposes; however, requests for data access may be submitted to the corresponding author and will be subject to institutional restrictions.
