# Supplementary figures and images for: Advancing Nursing Data Integration Through a Nursing Minimum Dataset for the Conceptual and Technical Development of a “Fall Prevention” Data Module: Development Study
Source: J Med Internet Res. 2026 Mar 17;28:e82417. doi: 10.2196/82417 (PMC13040164; doi:10.2196/82417)

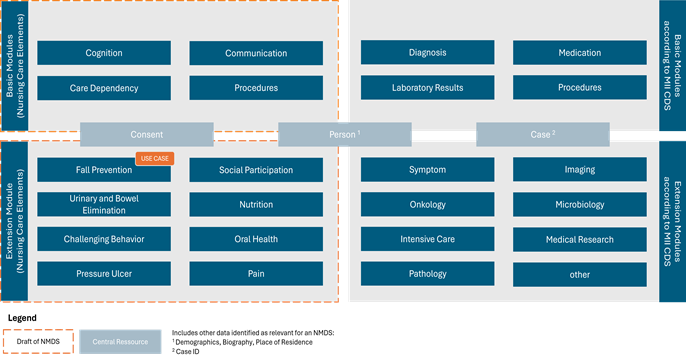

Supplement: Multimedia Appendix 1 [file jmir_v28i1e82417_app1.png]

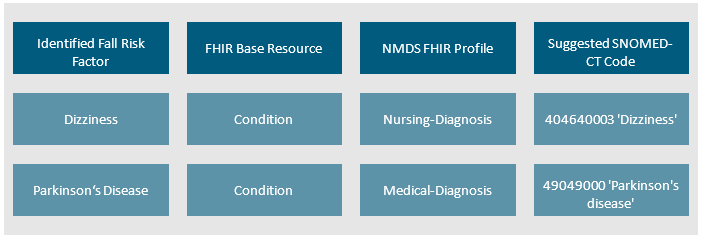

Supplement: Multimedia Appendix 3 [file jmir_v28i1e82417_app3.png]

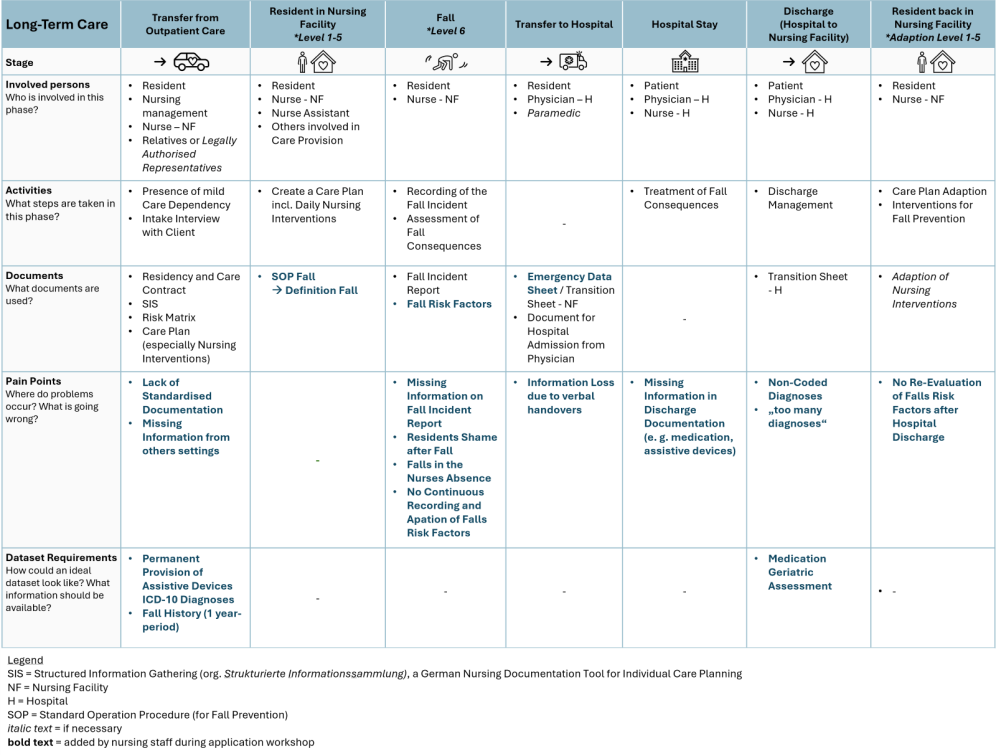

Supplement: Multimedia Appendix 4 [file jmir_v28i1e82417_app4.png]

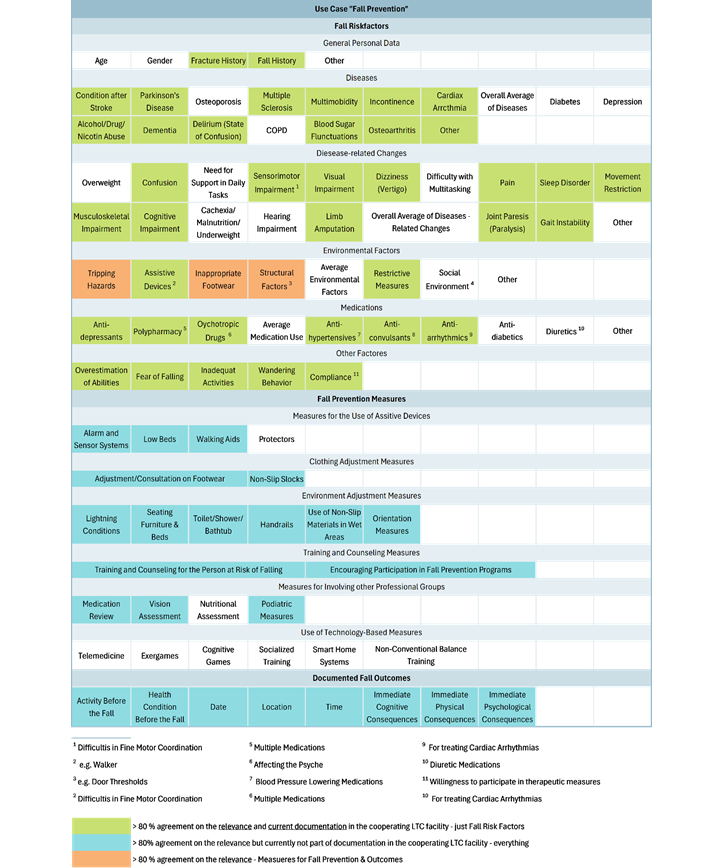

Supplement: Multimedia Appendix 5 [file jmir_v28i1e82417_app5.png]
