# Supplementary material for: Advancing Nursing Data Integration Through a Nursing Minimum Dataset for the Conceptual and Technical Development of a “Fall Prevention” Data Module: Development Study
Source: J Med Internet Res. 2026 Mar 17;28:e82417. doi: 10.2196/82417 (PMC13040164; doi:10.2196/82417)
Supplement: Multimedia Appendix 2 [file jmir_v28i1e82417_app2.pdf]

pflip-pflegedaten.de/umfrage

Core Data Set for Nursing Care

This survey is part of the PFLIP project funded by the German Federal Ministry of Education and Research (BMBF). For more information about the project, please visit **pflip-pflegedaten.de** or www.isst.fraunhofer.de/en/departments/healthcare/projects/PFLIP

If you have any questions, please contact us at pflip-umfrage@isst.fraunhofer.de

This is what it's all about:

https://youtu.be/3Nnvi-yYFJQ?feature=shared
(Embedded Video)

PFLIP is a project to develop a system that brings together care data from different institutions in order to gain new insights into care-related topics such as falls. We are a team from scientific institutions (University of Applied Sciences Bochum formerly University of Applied Sciences Health and Fraunhofer ISST) and practice partners (Diakonie Michaelshoven and Connext) who are collaborating on a research project.

This is what we want to find out:

1. What factors are decisive in determining the risk of falls among residents, patients, or customers?
1. What measures and information are important for better preventing falls in the future?

We will use the results of the survey to develop a system that anonymously collects information from various institutions in order to gain new insights into fall risks and fall prevention. Once the system is complete, this new knowledge can be used by caregivers in their everyday work.

- This questionnaire is **not a test** of your competence, qualifications, or work performance.
- Your **personal assessment** of falls based on your nursing experience is important to us. You have practical expertise on falls in everyday nursing care, and this experience is very interesting to us.
- The survey is **anonymous**. We cannot draw any conclusions about your identity. We will not share your answers with anyone. Your employer will not know whether you participated in the survey or what answers you gave.

By participating in this survey, you will help us with our research on fall prevention. This will enable us to support you as a caregiver in fall prevention with new knowledge in the future.

The survey takes about 30 minutes to complete, consists of four pages, and mainly consists of multiple-choice questions. The survey will be evaluated anonymously.

Yes, I have read the privacy policy and would like to participate

---

Scale 1 = important | rather important | rather unimportant | unimportant | no answer

Scale 2 = digitally documented | documented elsewhere | not documented | I don't know

FALL RISK FACTORS

We would like to hear about your experiences and professional assessments of risk factors for falls. This is about your **practical experience** and how you deal with these factors, not a test of your theoretical knowledge. Please indicate how important you consider the fall risk factors to be in **your everyday care work**.

Imagine you have to assess the risk of falling for a person in your facility. On the left side of the following tables, please indicate how important the points are for **assessing the risk of falling**. On the right side, enter how these points are documented in your daily care routine.

Question 1: General personal data Scale 1 + 2

- age
- gender
- history of falls
- history of fractures

Question 1* Free Text

You have indicated that a risk factor is not documented digitally. How is this risk factor documented in your organization? (e.g., file folder, sticky notes, etc.)

Question 1a Free Text

What other general personal risk factors for falls are there? (Only general factors, no illnesses/medications, etc.)

Question 2: Diseases Scale 1 + 2

- diabetes
- dementia
- COPD (chronic obstructive pulmonary disease)
- cardiac arrhythmia
- parkinson's disease
- multiple sclerosis (MS)
- post-stroke condition
- depression
- blood pressure fluctuations
- blood sugar fluctuations
- incontinence
- osteoporosis
- osteoarthritis
- alcohol/drug/nicotine abuse
- multimorbidity (multiple diseases at the same time)
- delirium (state of confusion)

Question 2* Free Text

You have indicated that a risk factor is not documented digitally. How is this risk factor documented in your organization? (e.g., file folder, notepad, etc.)

Question 2a Free Text

What other diseases can be risk factors for falls?

Question 3: Changes caused by illness Scale 1 + 2

- restricted movement
- sensorimotor impairment (difficulties with fine motor coordination)
- unsteady gait
- dizziness (vertigo)
- musculoskeletal impairment
- joint paresis (paralysis)
- visual impairment
- hearing impairment
- sleep disorder
- cognitive impairment
- confusion
- need for assistance with daily tasks
- difficulty multitasking
- cachexia/malnutrition/underweight
- overweight
- limb amputation
- pain

Question 3* Free Text

You have indicated that a risk factor is not documented digitally. How is this risk factor documented in your organization? (e.g., file folder, sticky notes, etc.)

Question 3a Free Text

What other disease-related risk factors for falls are there?

Question 4: Environment Scale 1 + 2

- tripping hazards
- assistive devices (e.g., walkers)
- unsuitable footwear
- measures that restrict freedom
- social environment (relatives, network)
- structural factors (e.g., door thresholds)

Question 4* Free Text

You have indicated that a risk factor is not documented digitally. How is this risk factor documented in your organization? (e.g., file folder, sticky notes, etc.)

Question 4a Free Text

What other environmental risk factors for falls might there be?

Question 5: Medications Scale 1 + 2

- multimedication
- anticonvulsants (to prevent epileptic seizures)
- psychotropic drugs (affect the psyche)
- antihypertensives (blood pressure lowering agents)
- antiarrhythmics (for the treatment of cardiac arrhythmias)
- sedatives (calming agents)
- antidepressants
- antidiabetics
- diuretics (urinary agents)

Question 5* Free Text

You have indicated that a risk factor is not documented digitally. How is this risk factor documented in your organization? (e.g., file folder, sticky notes, etc.)

Question 5a Free Text

What other medications (or groups of medications) can be risk factors for falls?

Question 6: Other Factors Scale 1 + 2

- self-overestimation
- inappropriate activities
- wandering behavior
- fear of falling
- compliance (willingness to cooperate with therapeutic measures)

Question 6* Free Text

You have indicated that a risk factor is not documented digitally. How is this risk factor documented in your organization? (e.g., file folder, sticky notes, etc.)

Question 7 Free Text

Are there any other risk factors for falling?

MEASURES

We would like to find out which nursing measures are particularly important. Again, this is about your personal assessment of the measures you take in your everyday nursing work.

Imagine that there is a person at risk of falling in your facility. How important do you consider the following nursing **measures** to be for preventing falls?

Please enter the importance of the measures on the left-hand side of the table. Then indicate on the right-hand side whether and how the information on the nursing measures is documented in your facility.

Question 1: Physical measures Scale 1 + 2

- physical exercises (e.g., strengthening, balance, endurance, and stretching exercises)
- balance exercises
- pain management
- monitoring of (side/interactive) effects of prescribed medication
- perception exercises (e.g. stimulation with essential oils)

Question 1a Free Text

You have specified a measure that is documented outside the care documentation system. Where or in which system is this measure documented in your organization?

Question 2: Measures for adjusting clothing Scale 1 + 2

- adjustment/advice on footwear (e.g., closed shoes, non-slip soles)
- anti-slip socks

Question 2a Free Text

You have specified a measure that is documented outside the care documentation system. Where or in which system is this measure documented in your organization?

Question 3: Measures for adapting the environment Scale 1 + 2

- checking/adjusting lighting conditions
- checking/adjusting seating furniture and beds
- checking/adjusting the toilet and shower/bathtub
- installing grab rails
- using non-slip materials in wet areas
- orientation measures (e.g., information signs, explanation and instruction regarding the premises)

Question 3a Free Text

You have specified a measure that is documented outside the care documentation system. Where or in which system is this measure documented in your organization?

Question 4: Training and consulting services Scale 1 + 2

- training and counseling measures for persons at risk of falling
- promoting participation in fall prevention programs for people at risk of falling

Question 4a Free Text

You have specified a measure that is documented outside the care documentation system. Where or in which system is this measure documented in your organization?

Question 5: Measures to involve other professional groups Scale 1 + 2

- review of medications (e.g., by pharmacologists /pharmacists, physicians)
- review of visual function (e.g., optometrist, ophthalmologist)
- review of nutrition
- podiatric measures (e.g., foot and ankle exercises, adjustment of footwear)

Question 5a Free Text

You have specified a measure that is documented outside the care documentation system. Where or in which system is this measure documented in your organization?

Question 6: Measures for the use of aids to prevent falls and injuries

- alarm and sensor systems (e.g., identification wristbands, bed alarm systems)
- low beds
- walking aids
- protective gear (e.g., hip protectors)

Question 6a Free Text

You have specified a measure that is documented outside the care documentation system. Where or in which system is this measure documented in your organization?

Question 7: Use of technology-based measures

- telemedicine (e.g., remote diagnostics)
- exergames (e.g., fitness games on consoles)
- cognitive games
- socialized training (e.g., virtual training via tablets in interaction with others)
- smart home systems (e.g., sensors for detecting falls)
- unconventional balance training (e.g., balance training with sensors worn on the body)

Question 7a Free Text

You have specified a measure that is documented outside the care documentation system. Where or in which system is this measure documented in your organization?

Question 8 Free Text

Are there any other nursing measures for fall prevention that you consider important?

CARE OUTCOMES AND DOCUMENTATION OF FALL EVENTS

Despite all precautions, falls can happen. Imagine that someone has fallen in your facility.

Please enter the points you consider important for documentation on the left side of the table. This is your **personal assessment** of the importance of each point.

On the right side of the table, indicate whether and how the information is documented in your facility.

Question 1: Information about the fall incident Scale 1 + 2

- date
- time
- location
- state of health immediately before the fall
- activity immediately before the fall
- immediate physical consequences of the fall (e.g., pain, wounds, changes in mobility, fractures)
- immediate cognitive consequences of the fall (e.g., changes in consciousness)
- immediate psychological consequences of the fall (e.g., feelings of insecurity, fear of falling),
- immediate follow-up measures (e.g., contact with a doctor, wound care)
- immediate evaluation of the fall risk factors of the person who fell
- immediate evaluation of the nursing measures taken to prevent falls in the person who fell

Question 1a Free Text

You have provided information that is documented outside the care documentation system. Where or in which system is this information about the fall documented? (e.g., fall log on paper)

Question 2 Free Text

Is there any other information that you consider important in connection with a fall?

YOUR PERSONAL DETAILS

Please fill in the following information about yourself.

We will anonymize your data and will not draw **any conclusions** about you personally. Your employer or others will not be informed by us whether you have participated or what answers you have given. We would like to use the following data to investigate how different groups (e.g., occupational groups, nursing staff from different regions, etc.) assess fall risks, measures, and outcomes.

This information is voluntary.

Question 1: How old are you? Number Input

Question 2: Please specify your gender Single Choice

- female
- male
- divers
- no answer

Question 3: What type of facility do you work in? Multiple Choice

- hospital
- rehabilitation facility
- inpatient long-term care
- nursing day care
- nursing night care
- short-term care
- outpatient nursing service
- assisted living facility
- psychiatric facility
- other facility (please specify)

Question 4: Please indicate which organization your institution is affiliated with Single Choice

- state institution
- church institution
- private institution
- no answer

Question 5: In which federal state are you employed? Single Choice

- Baden-Württemberg
- Bavaria
- Berlin
- Brandenburg
- Bremen
- Hamburg
- Hesse
- Mecklenburg-Western Pomerania
- Lower Saxony
- North Rhine-Westphalia
- Rhineland-Palatinate
- Saarland
- Saxony
- Saxony-Anhalt
- Schleswig-Holstein
- Thuringia
- no answer

Question 6: Please specify your qualifications Multiple Choice

- registered nurse (rn)
- registered nurse with additional training (rn+)
- nursing assistant/geriatric nursing assistant (lpn)
- nursing assistant
- unskilled assistant
- other qualification (please specify)

Question 7: Do you have any additional qualifications? Single Choice

- yes
- no
- no answer

Question 7a Free Text

Please list any additional qualifications you have.

Question 8: How much professional experience do you have? (in years) Number Input

Question 9: Are you involved in direct nursing care?

- yes
- no
- no answer

Question 10: Do you use software (e.g., Vivendi, Orbis, Nexus, etc.) for care documentation? If so, which one?

- no
- yes, I don't know what it's called.
- yes, it’s called xx
- no answer

--

Thank you for participating in our survey! Your practical expertise will help us better understand falls and support caregivers in fall prevention in the future.

If you have any comments, please feel free to note them here or contact us at pflip-umfrage@isst.fraunhofer.de.
